# Cholesterol Dietary Intake and Tumor Cell Homeostasis Drive Early Epithelial Tumorigenesis: A Potential Modelization of Early Prostate Tumorigenesis

**DOI:** 10.3390/cancers16112153

**Published:** 2024-06-06

**Authors:** Marine Vialat, Elissa Baabdaty, Amalia Trousson, Ayhan Kocer, Jean-Marc A. Lobaccaro, Silvère Baron, Laurent Morel, Cyrille de Joussineau

**Affiliations:** 1 GReD, CNRS UMR6293, Inserm U1103, Université Clermont Auvergne, 28 Place Henri Dunant, BP38, F63001 Clermont-Ferrand, France; marine.vialat@uca.fr (M.V.); elissa.baabdaty@uca.fr (E.B.); amalia.trousson@uca.fr (A.T.); ayhan.kocer@uca.fr (A.K.); j-marc.lobaccaro@uca.fr (J.-M.A.L.); silvere.baron@uca.fr (S.B.); laurent.morel@uca.fr (L.M.); 2 Groupe Cancer Clermont Auvergne, F63000 Clermont-Ferrand, France

**Keywords:** prostate cancer, cholesterol, early tumorigenesis, basal extrusion

## Abstract

**Simple Summary:**

Cholesterol and cholesterol derivatives accumulate in prostate cancer cells. Epidemiologic studies about diet and/or treatment with cholesterol-lowering drugs indicate that this metabolite plays a role in cancer progression, but they do not discriminate its possible role in cancer incidence. The goal of this study is so to determine whether cholesterol availability impacts tumor formation itself. Using a drosophila model specifically dedicated to the study of early epithelial tumorigenesis, we find that basal extrusion, a critical step of tumor formation, directly depends on cholesterol availability through dietary intake, and on cholesterol metabolization by the tumor cells. As we find that many genes related to cholesterol homeostasis and metabolism are ill-expressed in primary prostate cancer samples, this work indicates that cholesterol levels and metabolism could play a crucial role in the early phases of prostate cancer as well.

**Abstract:**

Epidemiological studies point to cholesterol as a possible key factor for both prostate cancer incidence and progression. It could represent a targetable metabolite as the most aggressive tumors also appear to be sensitive to therapies designed to decrease hypercholesterolemia, such as statins. However, it remains unknown whether and how cholesterol, through its dietary uptake and its metabolism, could be important for early tumorigenesis. Oncogene clonal induction in the *Drosophila melanogaster* accessory gland allows us to reproduce tumorigenesis from initiation to early progression, where tumor cells undergo basal extrusion to form extra-epithelial tumors. Here we show that these tumors accumulate lipids, and especially esterified cholesterol, as in human late carcinogenesis. Interestingly, a high-cholesterol diet has a limited effect on accessory gland tumorigenesis. On the contrary, cell-specific downregulation of cholesterol uptake, intracellular transport, or metabolic response impairs the formation of such tumors. Furthermore, in this context, a high-cholesterol diet suppresses this impairment. Interestingly, expression data from primary prostate cancer tissues indicate an early signature of redirection from cholesterol de novo synthesis to uptake. Taken together, these results reveal that during early tumorigenesis, tumor cells strongly increase their uptake and use of dietary cholesterol to specifically promote the step of basal extrusion. Hence, these results suggest the mechanism by which a reduction in dietary cholesterol could lower the risk and slow down the progression of prostate cancer.

## 1. Introduction

In the last few decades, prostate cancer (PCa) incidence has reached the first rank of men’s cancers. Among the various factors involved, dietary fat and especially cholesterol have been pointed out as potential modulators of prostate cancer risk and development [1,2].

In men, the association between cholesterol levels and aggressiveness has largely been reported [3,4,5,6,7] along with the correlation between cholesterol-lowering drugs, i.e., statins, and a decreased prostate cancer risk [7,8,9,10,11]. However, conflicting studies and meta-analyses have indicated that there is no clear association between plasma levels of total or fractions of cholesterol and prostate cancer (PCa) [5,12,13,14]. In the same way, the protective role of statins has recently been debated in regard to cancer progression [15,16], questioning the reality of the connection between plasma cholesterol levels and PCa. Nonetheless, cholesterol homeostasis and metabolism are indeed specifically altered in prostate tumors [17], and in many preclinical models, cholesterol has been linked to prostate cancer progression notably via its role as a lipid raft component promoting AKT and ERK signal transduction [18,19,20,21]. There, activation of cholesterol master regulators Liver X Receptors (LXRs) depletes cholesterol from lipid rafts and subsequently induces cancer cell apoptosis [22]. Furthermore, if a high-cholesterol diet does not alter murine prostate architecture by itself, the knockout of LXRs in this context induces prostatic intra-epithelial neoplasia [23], suggesting that loss of cholesterol homeostasis could also pave the way for prostate cancer development in the case of excessive cholesterol uptake.

We have previously developed an in vivo model of epithelial tumorigenesis in the accessory gland of drosophila melanogaster covering the steps from initiation to early progression characterized by tumor formation [24]. There, clonal expression of an oncogene in less than 1% of epithelial cells induces proliferation, loss of epithelial characteristics, migration, invasion, formation of tumors outside the gland, and even neo-tracheogenesis, an equivalent of neo-angiogenesis in drosophila, in these cells. Most importantly, co-expressing additional genetic constructs such as RNAi allows an in vivo understanding of the identity of the molecular mechanisms implicated in these largely understudied steps of early tumorigenesis. Indeed, drosophila represents a strong model for deciphering mechanisms related to epithelial cancers, such as lung or colon cancer [25,26]. The accessory gland itself represents a functional equivalent of a prostatic acinus [27,28,29,30,31], and in the last few years, it has been used as a model to decipher molecular signatures and molecular mechanisms related to prostate cancer [24,32,33,34,35,36]. Furthermore, there is a strong conservation of cholesterol’s role and metabolism in this insect [37,38,39]. Also, in mammals, cholesterol is supplied through diet and de novo synthesis [40], possibly masking the direct impact of dietary cholesterol on tumorigenesis. Crucially, the cholesterol auxotrophy of the fruit fly makes it solely dependent on dietary intake of this metabolite [41]. Together, this renders the drosophila accessory gland a particularly relevant model for assessing whether and how cholesterol could play a role on the early steps of epithelial tumorigenesis.

Here, we show that, in the accessory gland, tumors accumulate lipid droplets in which high amounts of cholesterol are present, as happens for cancer cells in human late-stage carcinogenesis [42]. In this condition, forcing further accumulation of cholesterol through genetic or dietetic approaches has no effect. On the contrary, tumor-cell-specific downregulation of cholesterol homeostasis decreases the critical step of basal extrusion, limiting the formation of tumors, in a cholesterol-dose-dependent manner. Finally, using a cohort of adenocarcinoma samples, we show that a profound deregulation of cholesterol homeostasis and metabolism occurs already in primary prostate cancer, suggesting that in humans, this deregulation could be important for the early steps of tumorigenesis.

## 2. Materials and Methods

### 2.1. Fly Stocks and Experimental Crosses

Flies allowing conditional clonal co-expression of GFP with oncogene Egfrλ were created, of the following genotype: y,w,HS:flp122/+;Act:FRTstopFRTGal4, UAS:GFP/CyO; UAS: EgfrλTop (coming from stock #59843). These flies were then crossed with the following stocks to realize experiments: UAS-GFP.nls (#4775, control condition, designed in figures as EGFRλ), UAS-LRP1 RNAi (#31151), UAS-LpR2 RNAi (#54461), UAS-Npc1a RNAi (#37504), UAS-Npc1b RNAi (#38296), UAS-CG8112 RNAi (#63035), and UAS-miR DHR96 (#27992, designed as UAS-DHR96 RNAi in figures) from the Bloomington Stock Center. 

### 2.2. Conditional Expression Induction

Briefly, a flippase (flp)/FRT system was activated by a 12 min heat-shock induction at 37 °C during the pupal stage to create an average of 4–6 cellular clones per accessory gland (representing ≈1% of total number of epithelial cells). Flies were then kept at 27 °C until the end of the pupal stage. Males were collected at emergence from pupae 3 to 3.5 days after heat shock and kept for another 2.5 days at 27 °C before dissection. For each cross, a minimum of 3 independent experiments were performed (N experiments), for a total of n pairs of accessory glands. In order to avoid possible individual bias, for each experiment, 100% of the males of the desired genotypes were dissected and processed through the different experimental procedures.

### 2.3. High-Cholesterol Diet 

After the cross, flies were either raised on a standard diet or high-cholesterol diet (HCD) complemented with 0.2% cholesterol (#3045, Sigma-Aldrich, Saint-Louis, MO, USA). These diet conditions were also maintained after the collection of males at the emergence from pupae.

### 2.4. Immunohistochemistry and Imaging

Accessory glands were dissected in PBS, fixed for 10 min in 4% formaldehyde, washed, and permeabilized for 15 min in PBS containing 0.2% Triton (PBS-T). Glands were blocked for 1 h with 0.5% of BSA in PBS-T then incubated overnight at 4 °C with primary antibodies diluted in the same blocking solution. After three washes in PBS-T, glands were incubated in secondary antibody diluted 1:1000 in blocking solution for 1 h at room temperature with DAPI (DiAminidoPhenylIndol, D8417, Sigma) 1:1000 (DNA staining) and/or Alexa633-phalloidin (A22284, Life Technology, Carlsbad, CA, USA) 1:5000 (to reveal F-actin). The glands were then washed three times with PBS and subsequently mounted in Vectashield (#-1000, Vector Laboratories, Newark, CA, USA). Imaging was realized with a Leica SP8 confocal microscope, and image stacks were processed either with ImageJ2 (version: 2.3.0/1.53t) or Imaris software (version 9.8.2).

The list of antibodies is as follows: Mouse Coracle (1:400, #C566.9 DSHB), NileRed (1ng/mL, Sigma-Aldrich, Saint-Louis, MO, USA), Bodipy (2ng/mL, #D3835 Invitrogen, Waltham, MA, USA), secondary antibodies coupled to different fluorophores 488 (1:1000, A11055 Invitrogen), Cy3 or Cy5 (1:1000, 711-165-152, 715-165-151, 715-175-150, Jackson Immunology, West Grove, PA, USA).

### 2.5. Clones, Cells, and Nuclei Size

Clones/tumors, cells, and nuclei volumes were determined from 3D reconstruction and automatic quantification with Imaris software. For each clone/tumor, the average cell size was determined by the ratio between the size of the clone and the number of nuclei in the considered clone.

### 2.6. Invasive Tumor Frequency

Tumor frequency was determined as the percentage of flies that displayed at least one tumor on their accessory glands at dissection.

### 2.7. RNA-seq Data

We retrieved processed RNA-seq data from the website http://cbio.mskcc.org/cancergenomics/prostate/data/ (now retrievable at https://www.cbioportal.org, Prostate Adenocarcinoma (MSK, Cancer Cell 2010), accessed on 2 June 2024). We only considered already treated and normalized log2 expression data.

### 2.8. Statistical Analyses

All experiments were repeated independently a minimum of three times (N: number of independent experiments) on numerous glands (n: number of pairs of glands or number of imaged and quantified glands for tumor size and nuclei number quantification). Statistical analyses were performed using GraphPad Prism 6. The tumor volumes as well as the numbers of nuclei and the volumes of droplets were compared by the Kruskal–Wallis test, while the percentages of glands presenting extraglandular tumors were compared by the Chi2 test. The human mRNA levels were compared using a two-tailed Mann–Witney test (for non-Gaussian data) or a two-sided unpaired *t* test (for Gaussian data).

## 3. Results

### 3.1. Accessory Gland Tumors Accumulate Cholesterol into Lipid Droplets

**Figure 1:**
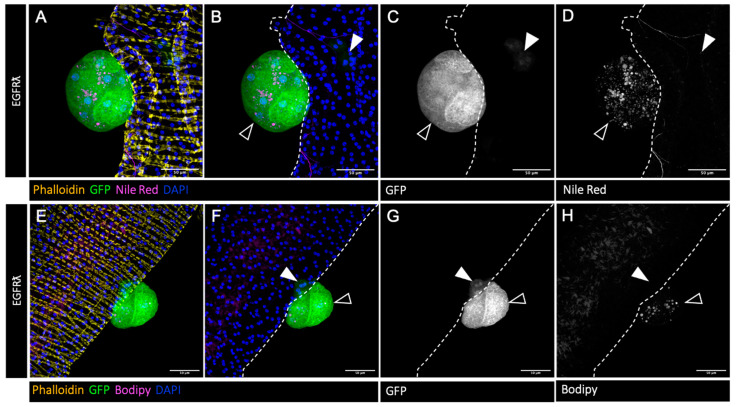
Drosophila accessory gland tumor cells accumulate cholesterol esters. (**A**–**H**) Confocal imaging of representative glands initiated for tumorigenesis. Clonal induction of an activated form of EGFR (EGRFλ) induces the formation of intraglandular clones (white arrowheads in (**B**–**D**) and (**F**–**H**)) and of tumors outside the gland (empty arrowheads in (**B**–**D**) and (**F**–**H**)). Muscle cells surrounding the glands are revealed by phalloidin staining of F-actin (yellow in (**A**,**E**)). Cell nuclei are revealed by DAPI staining (blue in (**A**,**B**) and (**E**,**F**)). Clonal cells are revealed by GFP co-expression (green in (**A**,**B**) and (**E**,**F**), grey for single channel in (**C**,**G**)). (**A**–**D**): Nile Red staining (magenta in (**A**,**B**) and grey in single channel (**D**)) shows an accumulation of neutral lipids specifically in tumor cells. (**E**–**H**) Bodipy staining (magenta in (**E**,**F**) and grey in single channel (**H**)) indicates a strong proportion of cholesterol esters among these neutral lipids. Representative images in (**A**–**H**) are from three or more experiments. Scale bars: 50 μM.

The accessory gland represents a perfectly defined epithelial compartment comparable to a single prostatic acinus. It is composed of a monolayer of secretory epithelial cells surrounded by a basement membrane in which a well-organized network of muscle fibers is completely enclosed (revealed by phalloidin staining in Figure 1A,D, yellow). Here, in order to mimic tumor initiation, clonal expression of constitutively active version of EGFR (EGFRλ condition) coupled to GFP was realized in approximatively 1% of the accessory gland cells. As previously described [24], this expression leads to the formation of two kinds of GFP-positive tumor cells (Figure 1): first, epithelial clones composed of slightly hypertrophic cells whose cytoskeletons are disorganized (Figure 1 white arrowheads), and second, GFP-positive tumors that grow outside the epithelial compartment as a result of a phenomenon of basal extrusion (Figure 1 empty arrowheads). These tumors, which represent a state of early progression, are composed of cells that bear hallmarks of cancer, such as hypertrophy, hyperplasia, and the loss of epithelial markers, and are furthermore associated with neotracheogenesis (a neoangiogensis equivalent) [24]. In this context, we wondered whether this parallel could be extended to abnormal lipid storage, a feature of prostate cancer cells [42]. Indeed, we observe by classical Nile Red staining the accumulation of intracellular neutral lipid droplets specifically in tumor cells (Figure 1A,B: magenta; Figure 1D: Nile Red staining only, grey). To further evaluate the content of these droplets, we used Bodipy, a marker of esterified cholesterol that indicates the presence of stored cholesterol into these droplets (Figure 1E,F: magenta; Figure 1H: Bodipy staining only, grey). So, we concluded that, as in humans, the formation of tumors during progression is accompanied by lipid, and especially esterified cholesterol, accumulation into the tumor cells.

### 3.2. Cholesterol Homeostasis Downregulation Impairs EGFRλ-Induced Cholesterol Storage

**Figure 2:**
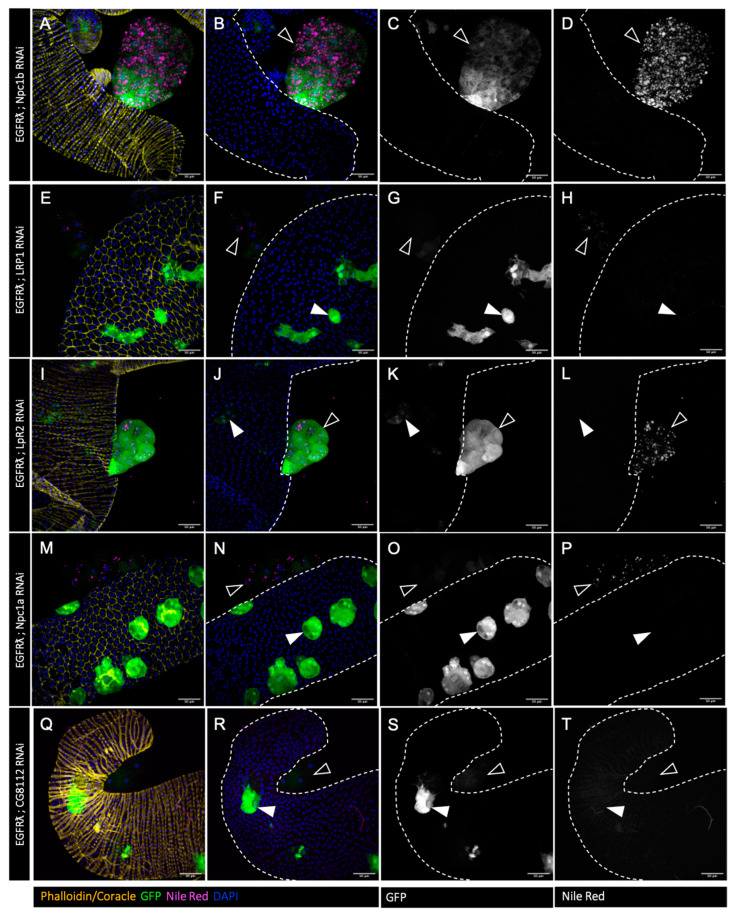
Tumor-specific downregulation of cholesterol uptake or metabolism reduces cholesterol ester accumulation. (**A**–**T**) Confocal imaging of representative glands initiated for tumorigenesis. White arrowheads indicate intra-glandular clones; empty arrowheads indicate tumors outside the gland. Muscle cells surrounding the glands are revealed by phalloidin staining in (**A**,**I**,**Q**) (yellow). Normal epithelial cells are revealed by coracle staining in (**E**,**M**) (yellow). Cell nuclei are revealed by DAPI staining (blue). Clonal cells are revealed by GFP co-expression (green in the first and second columns, grey in third). Neutral lipid accumulation is revealed by Nile Red staining (magenta in the first and second columns, grey in fourth). (**A**–**D**) Downregulation of ncp1b, which is expressed specifically in intestinal cells, is used as control RNAi. In this condition, a strong accumulation of neutral lipids is seen specifically in the tumor cells (in **A**,**B**,**D**) as for the EGFRλ condition. (**E**–**T**) Downregulation of genes implicated in cholesterol uptake (**E**–**L**), intracellular trafficking (**M**–**P**), or storage (**Q**–**T**) strongly impairs lipid accumulation (compare images in right column to (**D**)). Representative images in (**A**–**T**) from three or more experiments. Scale bars: 50 μm.

In order to test whether this excess of cholesterol is important for tumor formation, we then decided to downregulate the expression of genes involved in cholesterol uptake (LRP1, LpR2) (Figure 2E–H and I–L, respectively) [38], intracellular trafficking (Npc1a) (Figure 2M–P) [39,40], and storage (Figure 2Q–T) (CG8112—ortholog of SOAT1/ACAT1) [41] in a clone-specific manner. RNAi against Npc1b (Figure 2A–D), involved in cholesterol import, was used as a negative control as its expression is described as restricted to the gut [42]. In all the conditions, except Npc1b RNAi, we observed a strong decrease in lipid droplet volume and number (Figure 2), indicating that tumor-specific cholesterol storage is indeed impaired in the tumors.

We so concluded that each of these actors (except Npc1b) is necessary for cholesterol accumulation in drosophila accessory gland tumors, showing that all of them represent targetable proteins for the regulation of abnormal cholesterol accumulation.

### 3.3. Hyperactivation of Cell Autonomous Cholesterol Metabolism Specifically Drives Basal Extrusion

**Figure 3:**
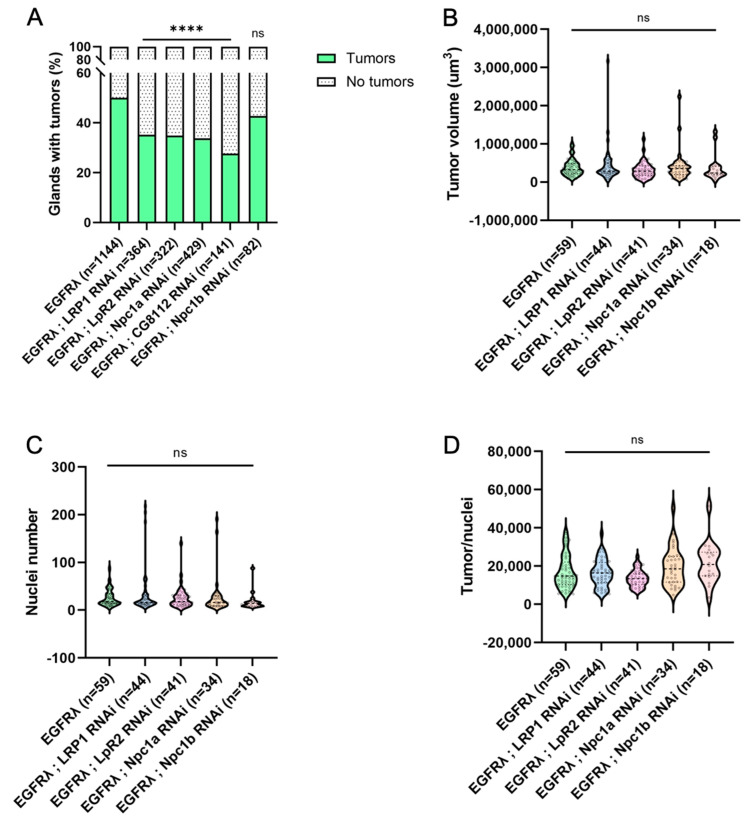
Tumor-specific downregulation of cholesterol uptake or metabolism impairs basal extrusion. (**A**) At dissection, the presence of tumors was assessed for all considered genotypes outside the glands for several flies (n) in independent experiments (N). As expected, expression of an RNAi against intestinal cell-specific Ncp1b does not induce a change in tumor formation compared to EGFRλ condition (right and left columns, respectively). On the contrary, downregulation of genes implicated in cholesterol uptake, intracellular trafficking, or storage significantly decreases tumor formation. (**B**–**D**) Tumor size (**B**), cell number (**C**), or tumor cell size remains unchanged when cholesterol uptake, intracellular trafficking, or storage is downregulated (see also Figure S1). (**A**) Chi2 test: EGFRλ: N = 29; LRP1 RNAi: N = 9; LpR2 RNAi: N = 8; Npc1a RNAi: N = 11; CG8112 RNAi: N = 5; Npc1b RNAi: N = 3. (**B**–**D**) Kruskal–Wallis tests: EGFRλ: N = 11; LRP1 RNAi: N = 10; LpR2 RNAi: N = 7; Npc1a RNAi: N = 8; Npc1b RNAi: N = 3. **** *p* < 0.0001; ns: non-significant.

We then assessed the percentage of glands bearing tumors in the previously described conditions. For all the genotypes, except the Npc1b RNAi negative control, we observed a significant decrease in tumor frequency (Figure 3A), showing that not only uptake (LRP1, LpR2) but also intracellular metabolism (Npc1a, CG8112) of cholesterol is necessary for tumor formation itself. This indicates that, in case of cell transformation by oncogene expression, a common cause of initiation, cholesterol metabolism is implicated in early tumorigenesis.

In order to see if cholesterol could be involved in early progression as well, we also characterized tumor volume (Figure 3B), tumor cell number (Figure 3C), and cell volume (Figure 3D) for all considered genotypes. Interestingly, all these parameters remain unaffected, and this phenotype is not altered throughout time (Figure S1).

Altogether, these results demonstrate that the observed deregulation of cholesterol homeostasis and metabolism in tumor cells does not impact tumor growth itself, but that it is necessary for tumor formation. So, we conclude that this apparent hyperactivation is specifically important for the early step of basal extrusion, when tumor cells actively leave the accessory gland to form tumors outside the epithelial compartment.

### 3.4. Further Cholesterol Accumulation Has No Effect on Early Tumorigenesis

**Figure 4:**
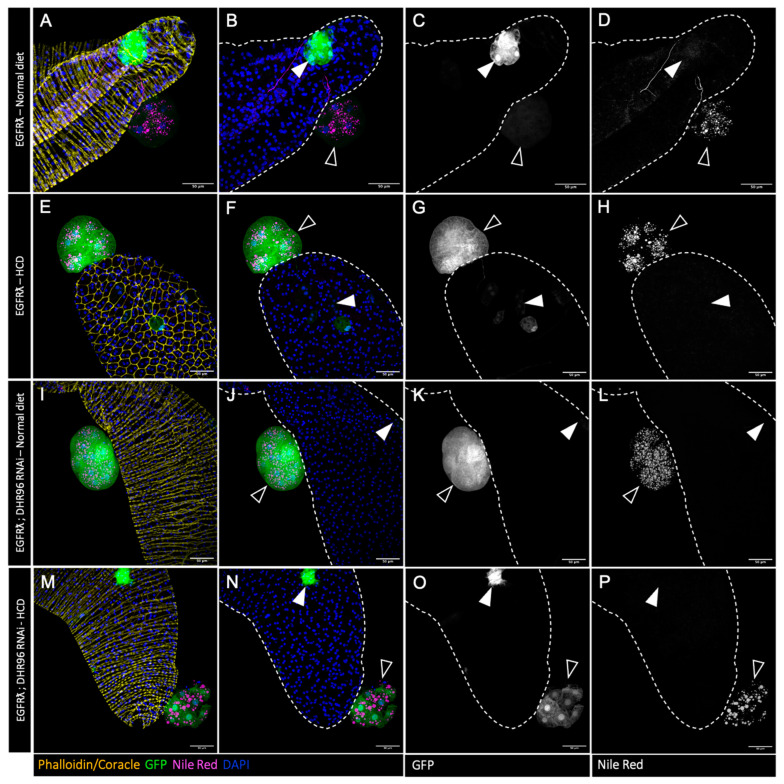
Further cholesterol homeostasis deregulation has no effect on tumor phenotype. (**A**–**P**) Confocal imaging of representative glands initiated for tumorigenesis. White arrowheads indicate intra-glandular clones; empty arrowheads indicate tumors outside the gland. Muscle cells surrounding the glands are revealed by phalloidin staining in (**A**,**I**,**M**) (yellow). Normal epithelial cells are revealed by coracle staining in (**E**) (yellow). Cell nuclei are revealed by DAPI staining (blue). Clonal cells are revealed by GFP co-expression (green in the first and second columns, grey in third). Neutral lipid accumulation is revealed by Nile Red staining (magenta in the first and second columns, grey in fourth). (**A**–**D**) Tumor control condition with normal diet. In this condition, a strong accumulation of neutral lipids is seen specifically in the tumor cells (in **A**,**B**,**D**). (**E**–**H**) High-cholesterol diet (+0.2% cholesterol, HCD) does not modify tumor phenotype despite slightly increased neutral lipid accumulation (quantification in Figure 5). (**I**–**P**) Downregulation of master controller of cholesterol homeostasis DHR96 +/− high-cholesterol diet (respectively normal diet in (**I**–**L**) and HCD in (**M**–**P**)) induces no evident changes in tumor phenotypes (quantifications in Figure 5). Representative images in (**A**–**P**) from three or more experiments. Scale bars: 50 μm.

As tumors accumulate high levels of cholesterol, we then wondered whether it was possible to push further this phenotype, and whether this could impact tumor characteristics or formation. In order to do so, we used two different strategies alone or combined (Figure 4). First, we mimicked the so-called Western diet, with flies fed either with a standard diet or a diet supplemented with 0.2% cholesterol (high-cholesterol diet condition or HCD). Second, in order to maximize tumor-cell-specific deregulation of cholesterol homeostasis, we downregulated the expression of the fly cholesterol sensor DHR96 [43,44].

**Figure 5:**
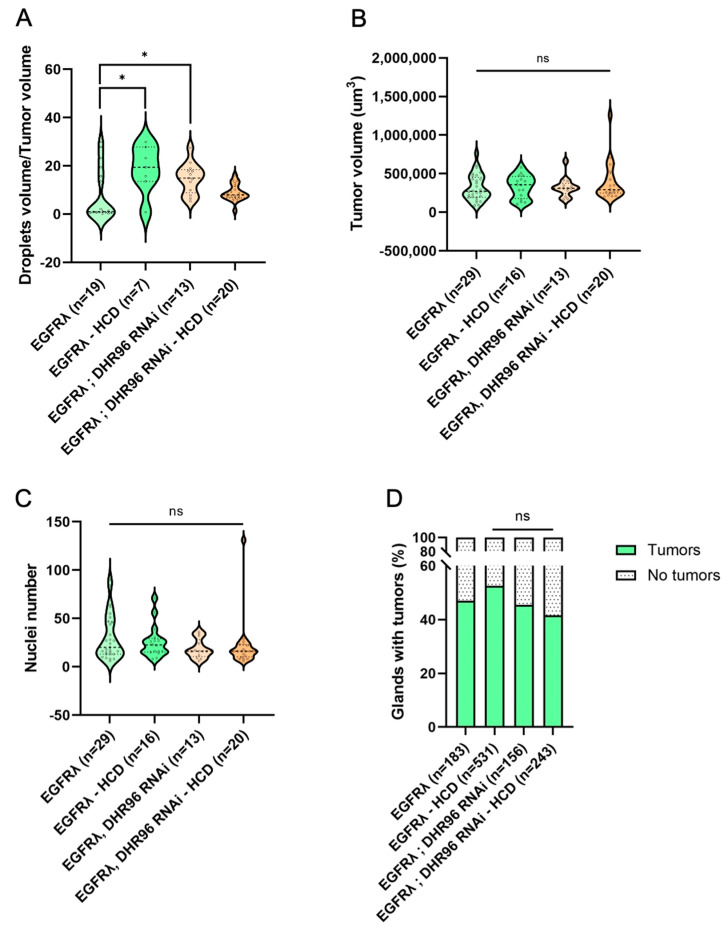
Forcing the accumulation of cholesterol further has a limited effect on tumor phenotype and no effect on basal extrusion. (**A**) High-cholesterol diet (HCD) or DHR96 tumor-cell-specific downregulation significantly increases the volume of lipid droplets compared to the EGFRλ condition. (**B**,**C**) Tumor size (**B**) and cell number (**C**) remain unchanged when flies are fed a high-cholesterol diet and/or when tumor cells are subjected to tumor-cell-specific downregulation of DHR96. (**D**) At dissection, the presence of tumors was assessed for all considered genotypes outside the glands for several flies (n) in several independent experiments (N). Neither HCD nor DHR96 downregulation induces a change in tumor formation compared to the EGFRλ condition. (**A**–**C**) Kruskal–Wallis test: EGFRλ: N = 6; EGFRλ—HCD: N = 6; DHR96 RNAi: N = 4; DHR96 RNAi—HCD: N = 3. (**D**) Chi2 test: EGFRλ: N = 9; EGFRλ—HCD: N = 10; DHR96 RNAi: N = 6; DHR96 RNAi—HCD: N = 6. * *p* < 0.05; ns: non-significant.

Compared to the tumor control condition (Nile Red staining in Figure 4A–D and quantification in Figure 5A), a high-cholesterol diet could slightly increase the accumulation of lipids into the tumors, denoting a limited capacity to exacerbate the abnormal lipid storage induced by the oncogene expression in the tumor cells. Interestingly, neither tumor characteristics (Figure 5B,C) nor basal extrusion (Figure 5D) were significantly affected by a high-cholesterol diet. We concluded that oncogene transformation is sufficient to obtain an independence from the diet for promoting tumor formation. For the deregulation of cholesterol homeostasis induced by the co-expression of DHR96 RNAi, a limited increase in lipid storage was observed (Figure 4I–L and quantification in Figure 5A), but once again, no effect on either tumor characteristics or tumor formation was observed (Figure 5B–D). We concluded that oncogene-driven deregulation of cholesterol homeostasis and metabolism may not exert a maximal effect in term of lipid accumulation, but is definitely on top for tumor promotion. Finally, double deregulation by downregulation of DHR96 associated with a high-cholesterol diet does not result in a higher accumulation of lipid droplets (Figure 4M–P and Figure 5A) and does not affect the studied parameters of tumorigenesis (Figure 5B–D) compared to EGFRλ conditions (see also Figure S1).

Overall, these results show that oncogene transformation exerts a profound deregulation of cholesterol homeostasis which in turn promotes basal extrusion, and that further deregulation through diet or targeting cholesterol regulators in the tumor cells cannot potentiate the initial effect due to the oncogene expression.

### 3.5. High-Cholesterol Diet Counteracts Effect of Cholesterol Metabolism Downregulation in EGFRλ-Induced Tumorigenesis

**Figure 6:**
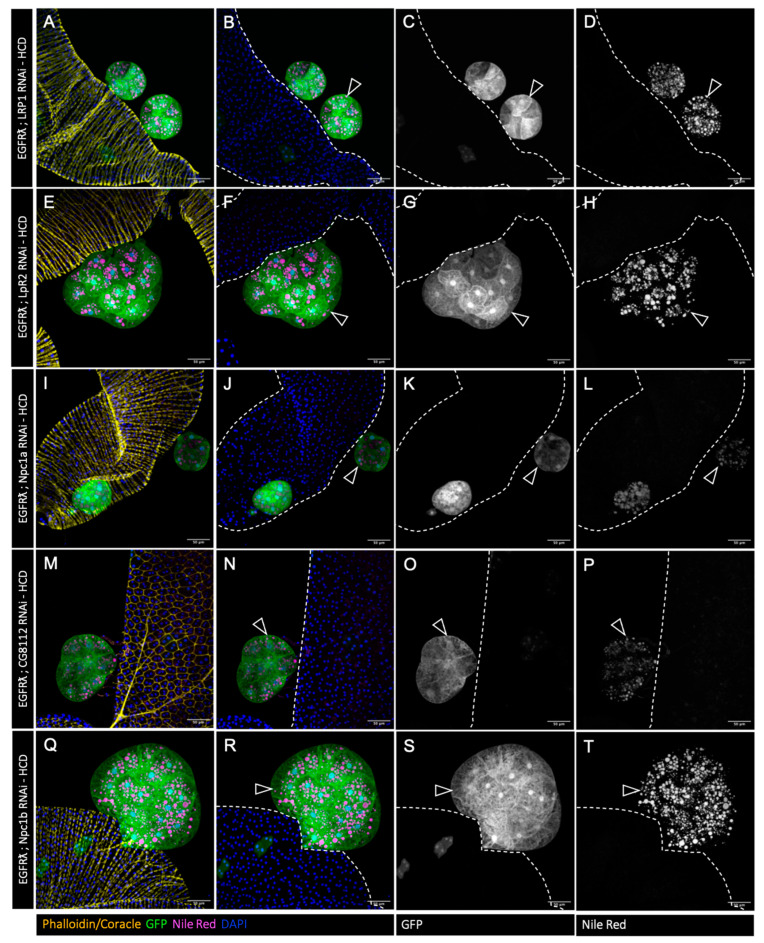
High-cholesterol diet restores tumor cell lipid accumulation but has no impact on tumor phenotype. (**A**–**T**) Confocal imaging of representative glands initiated for tumorigenesis. Empty arrowheads indicate tumors outside the gland. Muscle cells surrounding the glands are revealed by phalloidin staining in (**A**,**E**,**I**,**Q**) (yellow). Normal epithelial cells are revealed by coracle staining in (**M**) (yellow). Cell nuclei are revealed by DAPI staining (blue). Clonal cells are revealed by GFP co-expression (green in the first and second columns, grey in third). Neutral lipid accumulation is revealed by Nile Red staining (magenta in the first and second columns, grey in fourth). Increasing dietary cholesterol restores lipid accumulation in tumors harboring downregulation of cholesterol import (**A**–**H**), intracellular trafficking (**I**–**L**), or storage (**M**–**P**). Representative images in (**A**–**P**) from three or more experiments. Scale bars: 50 μm.

**Figure 7:**
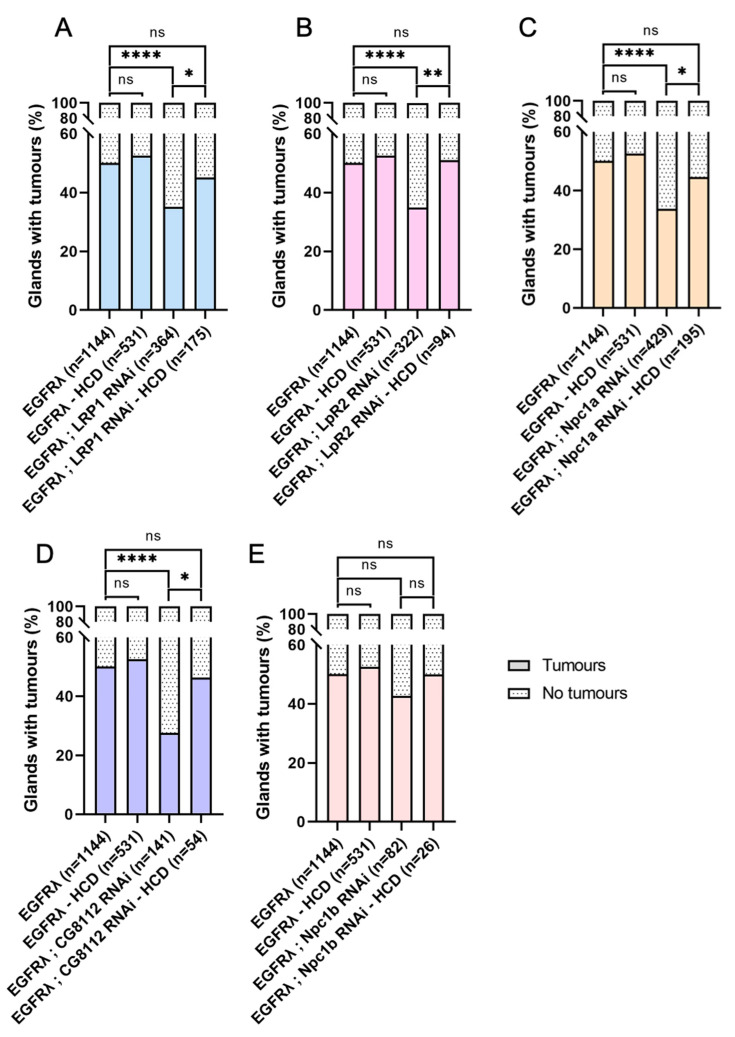
High-cholesterol diet counteracts the effect of decreased cholesterol uptake or metabolism on basal extrusion. At dissection, the presence of tumors was assessed for all considered genotypes outside the glands for several flies (n) in several independent experiments (N). (**A**–**D**) Increasing dietary cholesterol significantly increases tumor formation in tumors harboring downregulation of cholesterol import (**A**,**B**), intracellular trafficking (**C**), or storage (**D**) (compare the two right columns for each graph). By opposition, these high-cholesterol diet conditions become indistinguishable from the EGFRλ phenotype independently of diet status (compare right column to the two left columns). (**E**) As expected, a diet enriched in cholesterol has no effect on basal extrusion in a negative control condition, Npc1b RNAi. (**A**–**E**) Kruskal–Wallis tests: EGFRλ: N = 29; EGFRλ—HCD: 10; LRP1 RNAi: N = 9; LRP1 RNAi—HCD: N = 5; LpR2 RNAi: N = 8; LpR2 RNAi—HCD: N = 4; Npc1a RNAi: N = 11; Npc1a RNAi—HCD: N = 6; Npc1b RNAi: N = 3; Npc1b RNAi—HCD: N = 3; CG8112 RNAi: N = 5; CG8112 RNAi—HCD: N = 3. * *p* < 0.05; ** *p* < 0.01; **** *p* < 0.0001; ns: non-significant.

As the downregulation of cholesterol metabolism reduces basal extrusion, we then wondered if in this case, a high-cholesterol diet could now impact cholesterol storage and tumor formation (Figure 6). Interestingly, increasing dietary cholesterol does restore Nile Red staining in all genotypes (compare Figure 6A–P to Figure 2E–T). Furthermore, it completely counteracts the reduction in tumor formation that was observed in all the conditions where cholesterol metabolism is genetically impaired (Figure 7A–E). This indicates that dietary intake of cholesterol can finally play a major role in tumor formation in the context of decreased cholesterol access or metabolism by the tumor cells.

### 3.6. Genes Coding for Cholesterol Homeostasis and Metabolism Are Deregulated in Primary Prostate Cancer 

**Figure 8:**
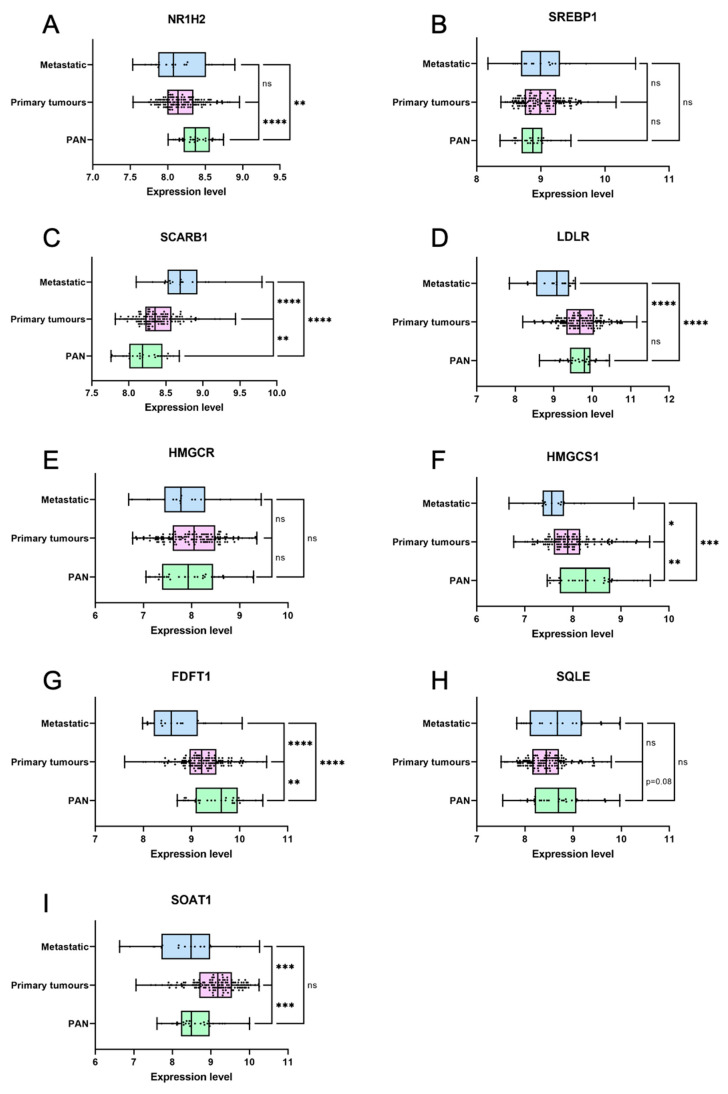
Cholesterol homeostasis and metabolism are deregulated in primary and metastatic prostate cancer. (**A**–**I**) Violin plots showing mRNA expression data for nine genes in normal prostate tissues (PAN, green), primary prostate tumors (primary tumors, pink), and metastatic prostate tumors (metastatic, blue). Expression data were first published by Taylor et al [46]. Unpaired t test: * *p* < 0.05; ** *p* < 0.01; *** *p* < 0.001; **** *p* < 0.0001; ns: non-significant. PAN: N = 29; primary tumors: N = 131; metastatic: N = 29.

In order to understand when cholesterol homeostasis/metabolism deregulation could be important during prostate carcinogenesis, we then determined from published data the expression of genes associated with this pathway in normal, primary, or metastatic samples [45]. First, master regulators of cell cholesterol homeostasis are already deregulated in primary cancer, with a decreased expression of *NR1H2*, coding for LXRβ, which is associated with the management of excess cholesterol (Figure 8A). On the contrary, *SREBF1*, which codes for SREBP1 whose role is to increase cholesterol cell levels, remains unchanged even though it tends to be upregulated (Figure 8B). This suggests an increased uptake and retention of cholesterol. Second, genes involved in cholesterol uptake such as *SCARB1*, coding for the receptor for high-density-lipoprotein (HDL) cholesterol, or *LDLR*, are either maintained or upregulated in primary cancer (Figure 8C,D). On the contrary, genes implicated in cholesterol de novo synthesis such as *HMGCR* (Figure 8E), *HMGCS1* (Figure 8F), *FDFT1* (squalene synthase, Figure 8G), and *SQLE* (Figure 8H) are either maintained or downregulated. This suggests a role of dietary uptake rather than de novo synthesis in the building of higher levels of cholesterol in the tumor cells. Third, *SOAT1*, coding for the enzyme that catalyzes cholesterol esterification, is specifically overexpressed in primary tumors compared to either normal or metastatic samples (Figure 8I). This suggests an early role of cholesterol accumulation in tumorigenesis.

Therefore, as cholesterol homeostasis is so clearly disturbed in primary cancer already, it could indeed promote the early steps of prostate tumorigenesis in addition to its role in later phases of progression.

Overall, we conclude that targeting cholesterol uptake and metabolism in the early phases of tumorigenesis could reduce tumor promotion, but that this effect is dependent on the level of cholesterol intake.

## 4. Discussion

Dietary habits, especially the Western diet characterized by a high content of lipids and sugar and a low level of vegetables, have been pointed out as a risk factor in a plethora of cancers. Prostate cancer follows the same trend, with some data associating cholesterol blood levels with cancer incidence and aggressiveness [1,2,43]. Experiments with high-cholesterol diets have been performed in murine models harboring human prostate cell line xenografts, indicating that the late evolution of cancer to castration resistance, possibly through higher androgen anabolism, correlates with high plasma cholesterol [44,45]. Also, cholesterol anabolism appears to increase in CRPC through *SQLE* overexpression, with an impact on lymph node invasion in a xenografted mouse model [47]. However, whether cholesterol metabolism could also be implicated in early tumorigenesis remains unknown. High plasma cholesterol levels have been linked to a risk of developing high-grade cancer [5,12], but the role of hypercholesterolemia in cancer incidence, if depicted in some clinical studies, is still not characterized by a meta-analysis approach [13]. Furthermore, the relative part of diet intake versus intra-tumoral production is not resolved. In this context, we have decided to use a drosophila model of early tumorigenesis in the accessory gland, a functional and structural equivalent of a prostatic acinus, previously developed by the team [24]. As insects are auxotrophs for cholesterol, we can directly evaluate the impact of normal and high-cholesterol diets on tumor incidence by modifying the food content.

In this context, we have first shown that tumors formed after basal extrusion in the gland accumulate lipids and especially esterified cholesterol into droplets, a phenomenon to compare with human pathology [42]. Neither this phenotype nor tumor incidence, or volume or cell number, are affected by a high-cholesterol diet. This shows that initiation, here by oncogene expression, is sufficient to render the tumor cells independent of normal or high-cholesterol diets. Indeed, lipid accumulation into droplets in tumor cells has never been associated with diet, but rather linked to genetic alterations appearing frequently in the PI3K/Akt pathway during the late stages of cancer progression [42]. However, this pathway is itself implicated in basal extrusion, and it could be recruited well before being affected by a genetic alteration, through an autocrine activation [24] for sustaining cholesterol accumulation and for promoting the early steps of cancer development. Overall, this could explain why, despite clear evidence of the impact of the Western diet on cancer incidence [1,43], the role of available cholesterol itself is still not forcefully established through its plasma levels [13].

We also decreased cholesterol homeostasis or metabolism specifically in tumor cells from initiation, by co-expression of RNAi targeting cholesterol import, transport, or storage along with the oncogene expression. There, a decrease in lipid accumulation was obtained, and if the average number of tumors was significantly decreased in these conditions, their size and cell composition remained mostly unaffected. This shows that the growth of the tumor itself does not depend much on hyperactivated cholesterol metabolism, but that, on the contrary, the formation of tumors outside the accessory gland does depend on it. This relies on the capacity for tumor cells to migrate through the basement membrane of the epithelial compartment, a phenomenon called epithelial basal extrusion. Basal extrusion is thought to be a funding event in adenocarcinoma formation [48,49], i.e., in a majority of cancers. However, despite its central role in carcinogenesis, this elusive event is largely understudied, due to the paucity of specific tools dedicated to its analysis, and logically to the absence of close-to-normal human samples where this event could be in progress. Overall, the molecular events that are known to be necessary for basal extrusion concern mostly the Ras/MAPK pathway [24,50,51,52], along with PI3K/Akt pathway co-activation [24]. Furthermore, it has also been shown that T-box transcription factors are necessary for basal extrusion [53], as well as the sphingosine 1-phosphate (S1P) pathway that could play a role as well in cell survival as in cell morphology [50,54]. Here, we add one component to the control of basal extrusion: hyperactivation of cholesterol metabolism. Then, basal extrusion appears more and more as a highly complex and highly controlled step of epithelial tumorigenesis. On the one hand, it provides a view of the richness of pathway deregulation appearing early in carcinogenesis, and on the other hand, it indicates potential targetable metabolites for limiting cancer progression.

If cholesterol accumulation seems critical for basal extrusion, it could also unveil more molecular mechanisms that are implicated in this step of tumorigenesis. Indeed, the role of cholesterol in human cancer has been linked to at least three different phenomena. First, it could just act as an energy provider: tumor cells exhibit a huge metabolism, and every lipid such as cholesterol is a highly caloric molecule. Second, it could act as a component of lipid rafts, and so as a molecule that increases the transduction response of pathways such as Ras/MAPK or PI3K/Akt/mTOR that have been hugely implicated in cancer promotion and progression [22,55]. Third, it could act as a precursor of steroid hormones, with androgen signaling being crucial for cancer progression and resistance as established for more than eighty years [56]. More work will have to be performed to understand if one or more of these mechanisms depend on cholesterol accumulation.

Concerning the source of cholesterol in tumor cells, by the use of an auxotroph model, we showed here that there is no need for intracellular cholesterol anabolism in order to acquire cholesterol accumulation in tumor cells (Figure 1). This correlates with the expression of genes in cancer patients, where cholesterol uptake genes such as *SCARB1* are overexpressed [17] and, in contrast, genes implicated in cholesterol anabolism are downregulated (Figure 8). If tumor cells are more dependent on dietary cholesterol than cell production, it could explain why the Western diet is a good indicator of prostate cancer development [38,39], and why statins, which block de novo synthesis of cholesterol, still have an uncertain role in prostate cancer incidence [13].

## 5. Conclusions

**Figure 9:**
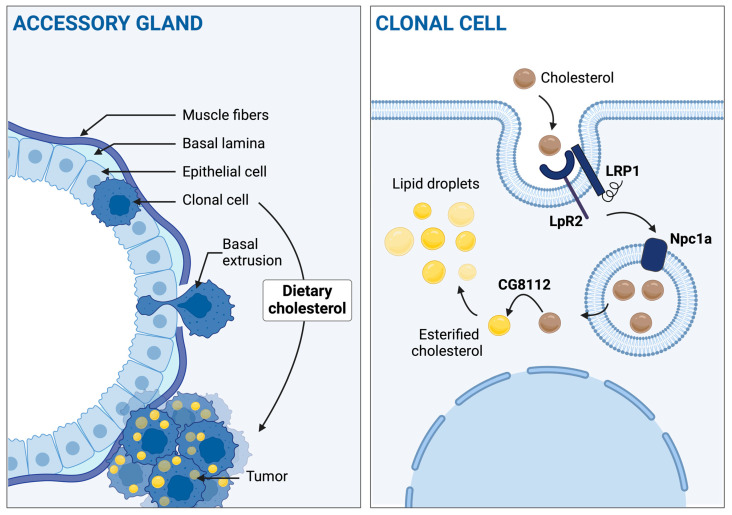
Dietary cholesterol and its metabolism and storage in situ are involved in basal extrusion. Left part, from top to bottom: After the oncogenic hit, clonal cells undergo basal extrusion to form tumors outside the gland, and these accumulate cholesterol into droplets. Dietary cholesterol promotes this phenomenon. Right part, from top to bottom: In situ cholesterol intake, trafficking, and storage drive the dietary cholesterol effect on tumorigenesis. Created with BioRender.com.

Altogether, this study highlights the pro-tumoral role of dietary cholesterol and its metabolism in situ in a model of prostate cancer and especially points out its role in the critical step of basal extrusion leading to the formation of tumors (Figure 9). Considering the strong deregulation of cholesterol homeostasis and metabolism in primary adenocarcinoma, as well as in other pathologies such as breast cancer [57], these findings could be indicative of the modus operandum by which high cholesterol metabolism could promote prostate carcinogenesis in humans. On the one hand, it brings yet another argument on the necessity to adopt a healthy diet to limit, amongst others, the risk of cancer. On the other hand, it points to a possible new step of tumorigenesis where cholesterol excess could be prejudiciable. In that regard, two future directions will have to be explored to better understand the significance of the present findings. First, for which mechanisms is this cholesterol accumulation necessary? Second, as drosophila has even recently shown its potential in precision medicine [58], it will be important to assess how much of these mechanisms are conserved in humans and thus how these findings could be useful for cancer care.

## Data Availability

The raw data supporting the conclusions of this article will be made available by the authors on request.

## Supplementary Materials

The following supporting information can be downloaded at https://www.mdpi.com/article/10.3390/cancers16112153/s1: Figure S1: Cholesterol dietary intake and/or cholesterol metabolism has no effect on tumor phenotype itself.

## References

[1] Shimizu H., Ross R.K., Bernstein L., Yatani R., Henderson B.E., Mack T.M. (1991). Cancers of the Prostate and Breast among Japanese and White Immigrants in Los Angeles County. Br. J. Cancer.

[2] Cook L.S., Goldoft M., Schwartz S.M., Weiss N.S. (1999). Incidence of Adenocarcinoma of the Prostate in Asian Immigrants to the United States and Their Descendants. J. Urol..

[3] Platz E.A., Clinton S.K., Giovannucci E. (2008). Association between Plasma Cholesterol and Prostate Cancer in the PSA Era. Int. J. Cancer.

[4] Platz E.A., Till C., Goodman P.J., Parnes H.L., Figg W.D., Albanes D., Neuhouser M.L., Klein E.A., Thompson I.M.J., Kristal A.R. (2009). Men with Low Serum Cholesterol Have a Lower Risk of High-Grade Prostate Cancer in the Placebo Arm of the Prostate Cancer Prevention Trial. Cancer Epidemiol. Biomark. Prev..

[5] Mondul A.M., Clipp S.L., Helzlsouer K.J., Platz E.A. (2010). Association between Plasma Total Cholesterol Concentration and Incident Prostate Cancer in the CLUE II Cohort. Cancer Causes Control.

[6] Kitahara C.M., Berrington de González A., Freedman N.D., Huxley R., Mok Y., Jee S.H., Samet J.M. (2011). Total Cholesterol and Cancer Risk in a Large Prospective Study in Korea. J. Clin. Oncol..

[7] Farwell W.R., D’Avolio L.W., Scranton R.E., Lawler E.V., Gaziano J.M. (2011). Statins and Prostate Cancer Diagnosis and Grade in a Veterans Population. J. Natl. Cancer Inst..

[8] Ukomadu C., Dutta A. (2003). Inhibition of Cdk2 Activating Phosphorylation by Mevastatin. J. Biol. Chem..

[9] Platz E.A., Leitzmann M.F., Visvanathan K., Rimm E.B., Stampfer M.J., Willett W.C., Giovannucci E. (2006). Statin Drugs and Risk of Advanced Prostate Cancer. J. Natl. Cancer Inst..

[10] Flick E.D., Habel L.A., Chan K.A., Van Den Eeden S.K., Quinn V.P., Haque R., Orav E.J., Seeger J.D., Sadler M.C., Quesenberry C.P.J. (2007). Statin Use and Risk of Prostate Cancer in the California Men’s Health Study Cohort. Cancer Epidemiol. Biomark. Prev..

[11] Shannon J., Tewoderos S., Garzotto M., Beer T.M., Derenick R., Palma A., Farris P.E. (2005). Statins and Prostate Cancer Risk: A Case-Control Study. Am. J. Epidemiol..

[12] Shafique K., McLoone P., Qureshi K., Leung H., Hart C., Morrison D.S. (2012). Cholesterol and the Risk of Grade-Specific Prostate Cancer Incidence: Evidence from Two Large Prospective Cohort Studies with up to 37 Years’ Follow Up. BMC Cancer.

[13] YuPeng L., YuXue Z., PengFei L., Cheng C., YaShuang Z., DaPeng L., Chen D. (2015). Cholesterol Levels in Blood and the Risk of Prostate Cancer: A Meta-Analysis of 14 Prospective Studies. Cancer Epidemiol. Biomark. Prev..

[14] Liu H., Shui I.M., Keum N., Shen X., Wu K., Clinton S.K., Cao Y., Song M., Zhang X., Platz E.A. (2023). Plasma Total Cholesterol Concentration and Risk of Higher-Grade Prostate Cancer: A Nested Case-Control Study and a Dose-Response Meta-Analysis. Int. J. Cancer.

[15] Meijer D., van Moorselaar R.J.A., Vis A.N., Bijnsdorp I. (2019). V Prostate Cancer Development Is Not Affected by Statin Use in Patients with Elevated PSA Levels. Cancers.

[16] Caro-Maldonado A., Camacho L., Zabala-Letona A., Torrano V., Fernández-Ruiz S., Zamacola-Bascaran K., Arreal L., Valcárcel-Jiménez L., Martín-Martín N., Flores J.M. (2018). Low-Dose Statin Treatment Increases Prostate Cancer Aggressiveness. Oncotarget.

[17] Celhay O., Bousset L., Guy L., Kemeny J.-L., Leoni V., Caccia C., Trousson A., Damon-Soubeyrant C., De Haze A., Sabourin L. (2019). Individual Comparison of Cholesterol Metabolism in Normal and TumourTumor Areas in Radical Prostatectomy Specimens from Patients with Prostate Cancer: Results of the CHOMECAP Study. Eur. Urol. Oncol..

[18] Zhuang L., Lin J., Lu M.L., Solomon K.R., Freeman M.R. (2002). Cholesterol-Rich Lipid Rafts Mediate Akt-Regulated Survival in Prostate Cancer Cells. Cancer Res..

[19] Patra S.K. (2008). Dissecting Lipid Raft Facilitated Cell Signaling Pathways in Cancer. Biochim. Biophys. Acta.

[20] Llaverias G., Danilo C., Wang Y., Witkiewicz A.K., Daumer K., Lisanti M.P., Frank P.G. (2010). A Western-Type Diet Accelerates Tumor Progression in an Autochthonous Mouse Model of Prostate Cancer. Am. J. Pathol..

[21] Oh H.Y., Lee E.J., Yoon S., Chung B.H., Cho K.S., Hong S.J. (2007). Cholesterol Level of Lipid Raft Microdomains Regulates Apoptotic Cell Death in Prostate Cancer Cells through EGFR-Mediated Akt and ERK Signal Transduction. Prostate.

[22] Pommier A.J.C., Alves G., Viennois E., Bernard S., Communal Y., Sion B., Marceau G., Damon C., Mouzat K., Caira F. (2010). Liver X Receptor Activation Downregulates AKT Survival Signaling in Lipid Rafts and Induces Apoptosis of Prostate Cancer Cells. Oncogene.

[23] Pommier A.J.C., Dufour J., Alves G., Viennois E., De Boussac H., Trousson A., Volle D.H., Caira F., Val P., Arnaud P. (2013). Liver x Receptors Protect from Development of Prostatic Intra-Epithelial Neoplasia in Mice. PLoS Genet..

[24] Rambur A., Lours-Calet C., Beaudoin C., Buñay J., Vialat M., Mirouse V., Trousson A., Renaud Y., Lobaccaro J.-M.A., Baron S. (2020). Sequential Ras/MAPK and PI3K/AKT/MTOR Pathways Recruitment Drives Basal Extrusion in the Prostate-like Gland of Drosophila. Nat. Commun..

[25] Levine B.D., Cagan R.L. (2016). Drosophila Lung Cancer Models Identify Trametinib plus Statin as Candidate Therapeutic. Cell Rep..

[26] Bangi E., Murgia C., Teague A.G.S., Sansom O.J., Cagan R.L. (2016). Functional Exploration of Colorectal Cancer Genomes Using Drosophila. Nat. Commun..

[27] Kalb J.M., DiBenedetto A.J., Wolfner M.F. (1993). Probing the Function of Drosophila Melanogaster Accessory Glands by Directed Cell Ablation. Proc. Natl. Acad. Sci. USA.

[28] Wolfner M.F. (1997). Tokens of Love: Functions and Regulation of Drosophila Male Accessory Gland Products. Insect Biochem. Mol. Biol..

[29] Rylett C.M., Walker M.J., Howell G.J., Shirras A.D., Isaac R.E. (2007). Male Accessory Glands of Drosophila Melanogaster Make a Secreted Angiotensin I-Converting Enzyme (ANCE), Suggesting a Role for the Peptide-Processing Enzyme in Seminal Fluid. J. Exp. Biol..

[30] Rewitz K.F., O’Connor M.B., Gilbert L.I. (2007). Molecular Evolution of the Insect Halloween Family of Cytochrome P450s: Phylogeny, Gene Organization and Functional Conservation. Insect Biochem. Mol. Biol..

[31] Sharma V., Pandey A.K., Kumar A., Misra S., Gupta H.P.K., Gupta S., Singh A., Buehner N.A., Ravi Ram K. (2017). Functional Male Accessory Glands and Fertility in Drosophila Require Novel Ecdysone Receptor. PLoS Genet..

[32] Molano-Fernández M., Hickson I.D., Herranz H. (2022). Cyclin E Overexpression in the Drosophila Accessory Gland Induces Tissue Dysplasia. Front. Cell Dev. Biol..

[33] Rambur A., Vialat M., Beaudoin C., Lours-Calet C., Lobaccaro J.-M., Baron S., Morel L., de Joussineau C. (2021). Drosophila Accessory Gland: A Complementary In Vivo Model to Bring New Insight to Prostate Cancer. Cells.

[34] Wilson C., Leiblich A., Goberdhan D.C.I., Hamdy F. (2017). The Drosophila Accessory Gland as a Model for Prostate Cancer and Other Pathologies. Current Topics in Developmental Biology.

[35] Ito S., Ueda T., Ueno A., Nakagawa H., Taniguchi H., Kayukawa N., Miki T. (2014). A Genetic Screen in Drosophila for Regulators of Human Prostate Cancer Progression. Biochem. Biophys. Res. Commun..

[36] Sekar A., Leiblich A., Wainwright S.M., Mendes C.C., Sarma D., Hellberg J.E.E.U., Gandy C., Goberdhan D.C.I., Hamdy F.C., Wilson C. (2023). Rbf/E2F1 Control Growth and Endoreplication via Steroid-Independent Ecdysone Receptor Signalling in Drosophila Prostate-like Secondary Cells. PLoS Genet..

[37] Danielsen E.T., Moeller M.E., Yamanaka N., Ou Q., Laursen J.M., Soenderholm C., Zhuo R., Phelps B., Tang K., Zeng J. (2016). A Drosophila Genome-Wide Screen Identifies Regulators of Steroid Hormone Production and Developmental Timing. Dev. Cell.

[38] Carvalho M., Schwudke D., Sampaio J.L., Palm W., Riezman I., Dey G., Gupta G.D., Mayor S., Riezman H., Shevchenko A. (2010). Survival Strategies of a Sterol Auxotroph. Development.

[39] Niwa R., Niwa Y.S. (2011). The Fruit Fly Drosophila Melanogaster as a Model System to Study Cholesterol Metabolism and Homeostasis. Cholesterol.

[40] Bloch K. (1965). The Biological Synthesis of Cholesterol. Science.

[41] Clark A.J., Block K. (1959). The Absence of Sterol Synthesis in Insects. J. Biol. Chem..

[42] Yue S., Li J., Lee S.-Y., Lee H.J., Shao T., Song B., Cheng L., Masterson T.A., Liu X., Ratliff T.L. (2014). Cholesteryl Ester Accumulation Induced by PTEN Loss and PI3K/AKT Activation Underlies Human Prostate Cancer Aggressiveness. Cell Metab..

[43] Haenszel W., Kurihara M. (1968). Studies of Japanese Migrants. I. Mortality from Cancer and Other Diseases among Japanese in the United States. J. Natl. Cancer Inst..

[44] Mostaghel E.A., Solomon K.R., Pelton K., Freeman M.R., Montgomery R.B. (2012). Impact of Circulating Cholesterol Levels on Growth and Intratumoral Androgen Concentration of Prostate Tumors. PLoS ONE.

[45] Moon H., Ruelcke J.E., Choi E., Sharpe L.J., Nassar Z.D., Bielefeldt-Ohmann H., Parat M.-O., Shah A., Francois M., Inder K.L. (2015). Diet-Induced Hypercholesterolemia Promotes Androgen-Independent Prostate Cancer Metastasis via IQGAP1 and Caveolin-1. Oncotarget.

[46] Taylor B.S., Schultz N., Hieronymus H., Gopalan A., Xiao Y., Carver B.S., Arora V.K., Kaushik P., Cerami E., Reva B. (2010). Integrative Genomic Profiling of Human Prostate Cancer. Cancer Cell.

[47] Xu Z., Huang L., Dai T., Pei X., Xia L., Zeng G., Ye M., Liu K., Zeng F., Han W. (2021). SQLE Mediates Metabolic Reprogramming to Promote LN Metastasis in Castration-Resistant Prostate Cancer. Onco. Targets Ther..

[48] Slattum G.M., Rosenblatt J. (2014). TumourTumor Cell Invasion: An Emerging Role for Basal Epithelial Cell Extrusion. Nat. Rev. Cancer.

[49] Fadul J., Rosenblatt J. (2018). The Forces and Fates of Extruding Cells. Curr. Opin. Cell Biol..

[50] Slattum G., Gu Y., Sabbadini R., Rosenblatt J. (2014). Autophagy in Oncogenic K-Ras Promotes Basal Extrusion of Epithelial Cells by Degrading S1P. Curr. Biol..

[51] Fadul J., Zulueta-Coarasa T., Slattum G.M., Redd N.M., Jin M.F., Redd M.J., Daetwyler S., Hedeen D., Huisken J., Rosenblatt J. (2021). KRas-Transformed Epithelia Cells Invade and Partially Dedifferentiate by Basal Cell Extrusion. Nat. Commun..

[52] Shirai T., Sekai M., Kozawa K., Sato N., Tanimura N., Kon S., Matsumoto T., Murakami T., Ito S., Tilston-Lunel A. (2022). Basal Extrusion of Single-Oncogenic Mutant Cells Induces Dome-like Structures with Altered Microenvironments. Cancer Sci..

[53] Shen J., Lu J., Sui L., Wang D., Yin M., Hoffmann I., Legler A., Pflugfelder G.O. (2014). The Orthologous Tbx Transcription Factors Omb and TBX2 Induce Epithelial Cell Migration and Extrusion in Vivo without Involvement of Matrix Metalloproteinases. Oncotarget.

[54] Hendley A.M., Wang Y.J., Polireddy K., Alsina J., Ahmed I., Lafaro K.J., Zhang H., Roy N., Savidge S.G., Cao Y. (2016). P120 Catenin Suppresses Basal Epithelial Cell Extrusion in Invasive Pancreatic Neoplasia. Cancer Res..

[55] Zhuang L., Kim J., Adam R.M., Solomon K.R., Freeman M.R. (2005). Cholesterol Targeting Alters Lipid Raft Composition and Cell Survival in Prostate Cancer Cells and Xenografts. J. Clin. Investig..

[56] Huggins C., Hodges C. (1941). V Studies on Prostatic Cancer. I. The Effect of Castration, of Estrogen and of Androgen Injection on Serum Phosphatases in Metastatic Carcinoma of the Prostate. Cancer Res..

[57] Graziani S.R., Igreja F.A.F., Hegg R., Meneghetti C., Brandizzi L.I., Barboza R., Amâncio R.F., Pinotti J.A., Maranhão R.C. (2002). Uptake of a Cholesterol-Rich Emulsion by Breast Cancer. Gynecol. Oncol..

[58] Bangi E., Ang C., Smibert P., Uzilov A.V., Teague A.G., Antipin Y., Chen R., Hecht C., Gruszczynski N., Yon W.J. (2019). A Personalized Platform Identifies Trametinib plus Zoledronate for a Patient with KRAS-Mutant Metastatic Colorectal Cancer. Sci. Adv..

