# Supplementary material for: Cholesterol Dietary Intake and Tumor Cell Homeostasis Drive Early Epithelial Tumorigenesis: A Potential Modelization of Early Prostate Tumorigenesis
Source: Cancers (Basel). 2024 Jun 6;16(11):2153. doi: 10.3390/cancers16112153 (PMC11172085; doi:10.3390/cancers16112153)
Supplement: Supplementary file 1 [file cancers-16-02153-s001.zip › cancers-2997304-supplementary.pdf]

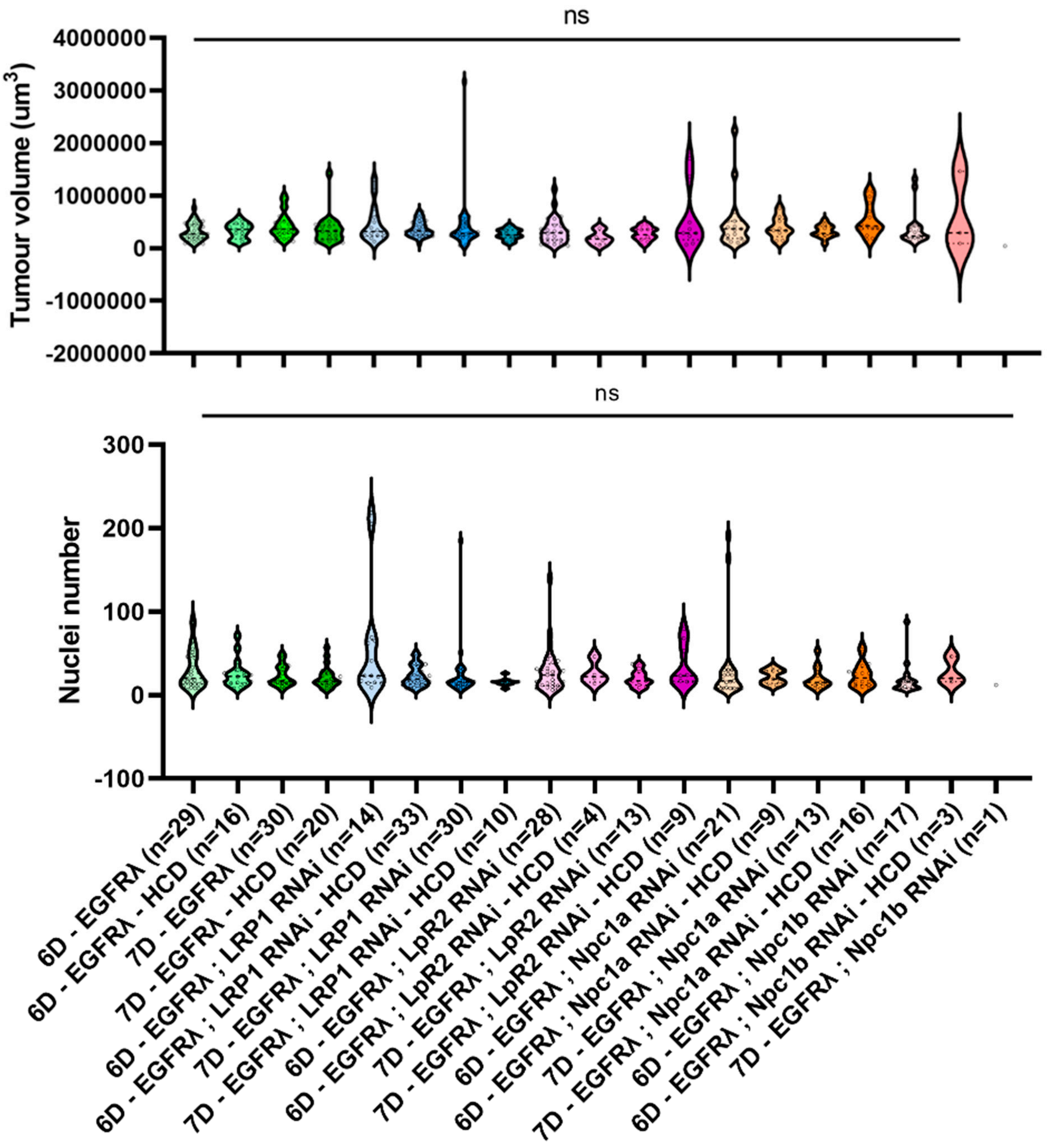

Figure S1. Cholesterol dietary intake and/or downregulation of cholesterol uptake or metabolism has no effect on tumour phenotype itself.

Increasing dietary cholesterol (HCD condition) and/or downregulation of cholesterol import, intracellular trafficking or storage has no effect on the volume and the number of cells of extra-epithelial tumours.
